# Mortality as an indicator of patient safety in orthopaedics: lessons from qualitative analysis of a database of medical errors

**DOI:** 10.1186/1471-2474-13-93

**Published:** 2012-06-08

**Authors:** Sukhmeet S Panesar, Andrew Carson-Stevens, Bhupinder S Mann, Mohit Bhandari, Rajan Madhok

**Affiliations:** 1 Department of Surgery and Cancer, St Mary's Hospital, Imperial College London, Praed Street, London, W2 1NY, UK; 2 Centre for Population Health Sciences, The University of Edinburgh, Medical School, Teviot Place, Edinburgh, EH8 9AG, UK; 3 National Patient Safety Agency, 4-8 Maple Street, London, W1T 5HD, UK; 4 Department of Primary Care and Public Health, Cardiff University, 2nd Floor, Neuadd Meirionnydd, Heath Park, Cardiff, CF14 4YS, UK; 5 Southmead Hospital and Avon Orthopaedic Centre, North Bristol NHS Trust, Westbury-on-Trym, Bristol, BS10 5NB, UK; 6 Center for Evidence-Based Orthopaedics, McMaster University, Department of Orthopaedic Surgery, McMaster University, 293 Wellington Street North, Suite 110, Hamilton, ON, L8S4L8, Canada; 7 Parkway Business Centre, NHS Manchester, Parkway 3, Princess Road, Manchester, M14 7 LU, UK

**Keywords:** Patient safety, Errors, Orthopaedics, Trauma surgery, Quality improvement

## Abstract

**Background:**

Orthopaedic surgery is a high-risk specialty in which errors will undoubtedly occur. Patient safety incidents can yield valuable information to generate solutions and prevent future cases of avoidable harm. The aim of this study was to understand the causative factors leading to all unnecessary deaths in orthopaedics and trauma surgery reported to the National Patient Safety Agency (NPSA) over a four-year period (2005–2009), using a qualitative approach.

**Methods:**

Reports made to the NPSA are categorised and stored in the database as free-text data. A search was undertaken to identify the cases of all-cause mortality in orthopaedic and trauma surgery, and the free-text elements were used for thematic analysis. Descriptive statistics were calculated based on the incidents reported. This included presenting the number of times categories of incidents had the same or similar response. Superordinate and subordinate categories were created.

**Results:**

A total of 257 incident reports were analysed. Four main thematic categories emerged. These were: (1) stages of the surgical journey – 118/191 (62%) of deaths occurred in the post-operative phase; (2) causes of patient deaths – 32% were related to severe infections; (3) reported quality of medical interventions – 65% of patients experienced minimal or delayed treatment; (4) skills of healthcare professionals – 44% of deaths had a failure in non-technical skills.

**Conclusions:**

Most complications in orthopaedic surgery can be dealt with adequately, provided they are anticipated and that risk-reduction strategies are instituted. Surgeons take pride in the precision of operative techniques; perhaps it is time to enshrine the multimodal tools available to ensure safer patient care.

## Background

Healthcare is a risky business with adequate attention to patient safety being paid only in the last decade or so. This is in contrast to the aviation industry which has focused on building safe systems since World War II. The Institute of Medicine (IOM) published a sentinel report, *To Err is Human* followed by *Crossing the Quality Chasm,* almost a decade ago [1,2]. The former portrayed medical error as a key challenge for public health. The limited appreciation that the lethality of error was as high as breast cancer or road traffic accidents meant the challenge of dealing with this problem was significant. Domestic and international health policy has since prioritised the importance of reducing the burden of iatrogenic harm; especially through the work of the World Health Organization’s (WHO) World Alliance for Patient Safety [3].

The creation of patient safety reporting systems (PSRSs) has been a key component and signals a significant commitment to learning from safety incidents and promoting patient safety internationally. The largest such system, with over 5.5 million reported cases of iatrogenic harm from 2005–2009, is housed at the National Patient Safety Agency (NPSA) [4]. The NPSA’s National Reporting and Learning System (NRLS) is a voluntary, national reporting system set up in 2003 for the National Health Service (NHS) in England and Wales. It is one of the largest and most comprehensive reporting systems in the world and reporting to the NRLS has increased year-on-year [5]. All staff working within the NHS can report incidents through their parent institution to ensure local action can be taken when needed. A representative from each parent institution is responsible for uploading records to the national database. In addition, healthcare staff, patients and members of the public can report incidents independently through the NRLS website [6].

Each NRLS report refers to an unintended or unexpected incident that could have or did lead to harm for one or more patients receiving NHS-funded care. It includes the reporting of those incidents which did not lead to harm, i.e. where an error took place but it did not harm the patient, or where an incident was prevented from reaching the patient. These incidents are further stratified into different levels of harm [5]. In order to integrate the wide variety of local safety management systems and software, the NRLS has 75 data fields, including incident categories at two levels, specialty and location of the incident, and free-text descriptions of the events [7]. Each incident reported as leading to death or serious harm is reviewed individually by trained clinical staff and a range of outputs are produced to provide solutions to patient safety problems. These include one-page reports called *Rapid Response Reports*, quarterly data summaries and topic-specific information on topics such as preventing inpatient falls in hospitals. There is constant consultation with subject-matter experts, including professional organisations such as the Royal College of Surgeons. NHS organisations also have deadlines imposed on them regarding when they should have implemented any findings from these reports [5].

Reports continue to accrue at an accelerating rate, with the database currently receiving approximately one-quarter of a million reports per quarter. Data from 2008 revealed that approximately 152,017 incidents (15.5%) related to surgery each quarter and, of these, 32.4% (49,254 incidents) were from orthopaedic and trauma surgery [8]. During the same period, 5,258,594 finished consultant episodes occurred in surgery and, of these, 1,144,520 were in the specialty of orthopaedics [9].

Despite recent attention, patient safety is not a new or a novel concept. In fact, the process of reviewing clinical outcomes in a standardised fashion began in parallel with the rise of the modern teaching hospital. The practice was refined through the work of Ernest Amory Codman, a surgeon at Massachusetts General Hospital in the early 20th century, who developed the ‘end result system’ [10]. He detailed the clinical history and outcomes of each of his patients on a set of cards and used this information to review adverse events systematically and to categorise their precedent errors. This was the precursor to the modern day morbidity and mortality (M&M) meetings in surgery. In tandem with active steps taken to introduce these meetings as part of surgical training in the USA, the Royal College of Surgeons of England demanded each hospital should hold regular M&M meetings in order to receive recognition for the training of junior surgical staff [11]. The Confidential Enquiry into Peri-operative Deaths focused attention on the importance of identifying deficiencies in standards of care; in addition, increasing litigation with expensive settlements provided an added stimulus to avoid problems caused by poor management or negligence [12]. Clearly there are lessons to be learnt by studying mortality reported to a national database of incidents.

The aim of this study was to understand the causative factors leading to potentially avoidable deaths (mortality) in orthopaedics and trauma surgery reported to the NPSA over a four-year period (2005–2009), using a qualitative approach. It is anticipated that the analysis of longitudinal data will generate discussion about the utility and value of reporting adverse incidents. More importantly, that it will inform the development of appropriate interventions to reduce avoidable harm.

## Methods

### Study design and data collection

Data from the NRLS database were analysed for all incidents reported in the specialty of trauma and orthopaedics between January 2005 and December 2009. The structure of the NRLS has been described previously [4]. The domains searched were ‘acute/general hospital’ and ‘trauma and orthopaedics’, and the search was limited to England. Cases identified as ‘deaths’ were selected. Data was abstracted onto a data collection sheet designed *a priori.* Incidents of non-fatal harm were excluded to minimise subjectivity bias, as there is no agreed standard definition for such incidents.

Two hundred and fifty seven (257) incident reports were analysed and subjected to thematic content analysis. The analysis generated thematic (superordinate) categories. The mean inter-rater reliability (Kappa) across all categories was 0.74 (SD 0.27).

### Justification of method

Qualitative research methods were used in order to garner an understanding of the causes of these deaths. In qualitative research, little is known about the problem or influencing variables prior to study [13]. A thematic analysis was appropriate since there are no known factors available to relate the cause of deaths in orthopaedics and trauma patients. This process involves categorising data through the development of a thematic framework by identifying and summarising key themes.

### Data analysis

Data from the NRLS, specifically the free-text elements, were analysed thematically by using the constant comparative method [14]. This was primarily conducted by two of the co-authors (SSP and ACS), with additional input from the other co-authors. Cases and incident reports from the database were extracted and considered as units of analysis for the present study. Each incident report was allocated a unique identification number in order to specify which responses corresponded to the certain unit in the study, and to protect the identities and confidentiality of those involved in the cases reviewed. Familiarisation was achieved by both researchers repeatedly reading the free-text reports.

A thematic framework was developed by generating thematic categories to form superordinate categories that grouped themes together. Subordinate categories were also created that broke themes down for greater granularity.

In essence, free text was read to identify common and recurrent themes. Items of data were repeatedly compared from the dataset and categories were defined in relation to each other. Subsequently, salient issues and key themes emerged. This ensured that themes, differences and relations between categories were re-examined and confirmed or modified [15]. For the specific method in analysing the data, thematic analysis was used to determine whether there were certain concepts present in texts or written documents [16]. The purpose of determining themes and concepts within documentation or texts is to permit the investigator to quantify and analyse the data such that inferences about the written text may be made. To conduct a thematic analysis on the text that was recorded from the responses, the responses were coded into manageable categories on a variety of levels [17]; this included breaking the responses down into key components, words, sentences or themes. These themes or key components were then examined using relational analysis to determine whether there were any relationships between the reports.

Indexing was achieved by coding each line of the free-text according to the thematic framework. ACS checked reliability by indexing a third of the reports. In cases of any discrepancies that existed between these two authors in their classification of free text, consensus was achieved by direct discussion and re-definition of categories agreed. The final coding framework applied to the reports was agreed by SSP and ACS.

From the results of the thematic analysis, descriptive statistics were calculated based on the incidents reported. This included presenting the number of times the incident reports had the same or similar response. The percentage or proportion of incidents with a particular response was then calculated based on the numbers calculated in the frequency distribution.

Microsoft Excel was used to organise the themes and trends of the information generated from the incident reports.

## Results

**Figure 1:**
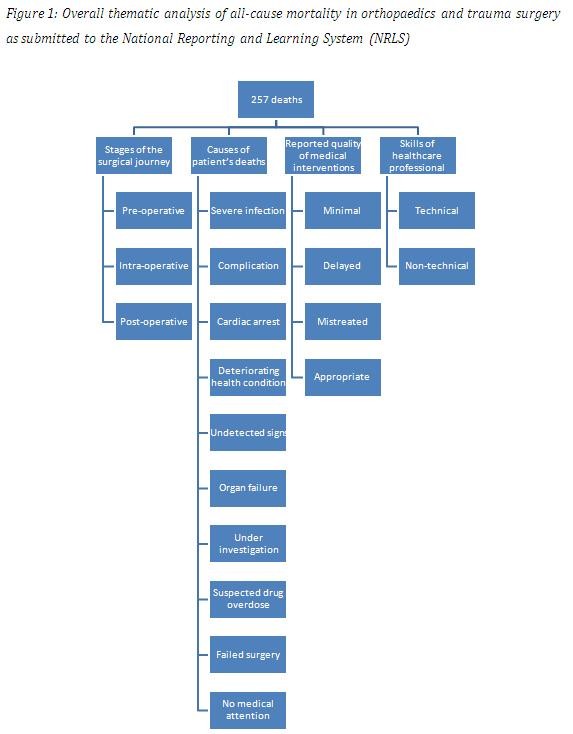
Thematic analysis of all-cause mortality.

Two hundred and fifty seven (257) incident reports were analysed and subjected to thematic content analysis. The analysis generated four thematic (superordinate) categories: (1) stages of the surgical journey; (2) causes of patient deaths; (3) reported quality of medical interventions; (4) skills of healthcare professionals. Superordinate categories were broken down into subordinate groups, as shown in Figure 1. The mean inter-rater reliability (Kappa) across all categories was 0.74 (SD 0.27).

### The surgical journey

This referred to three distinct phases of the journey that patients underwent when undergoing an operation: pre-operative, peri-operative and post-operative stages. 191/257 (74.3%) incidents had enough information to generate thematic analysis in this section. Of these, 118 (61.7%) deaths were in the post-operative surgical period; 45 (23.5%) were during the pre-operative phase; whilst 28 reported death peri-operatively. The incidents that could not be analysed (66/257, 25.7%) ranged from brief reports where no phase could be identified as they consisted of a few words (for example *‘patient fell’* or *‘bleeped surgeon, no response’*), to detailed reports which could have occurred in any phase (for example *‘patient deteriorated, surgical team alerted and resuscitation commenced’)*.

### Causes of patient deaths

In the examination of the causes of death reflected in the incident reports, 193 incidents revealed 10 causes of patient death, as shown in Table 1.

**Table 1:** Causes of patient deaths

**Categorical Variable**	**# of Incidents**	**% of Incidents**
Severe Infection	62	32.12
Surgical Complications	36	18.65
Cardiac Arrest	34	17.62
Deteriorating health condition	22	11.40
Undetected signs	15	7.77
Organ failure	8	4.14
Under investigation	5	2.59
Suspected drug overdose	5	2.59
Failed surgery	4	2.07
No medical attention	2	1.04

Whilst we identified 10 causes of accidental death, severe infection was consistently indicated as the leading cause of death. The data in this group were categorised further: Clostridium difficile (C. diff; 69.8%), wound infection (12.7%), septic shock (6.3%), blood culture bacteria (2%) and ‘other’ infections.

### Quality of medical interventions

Of the 257 reviewed cases, only 126 cases reported the incident in sufficient detail to assess the quality of medical intervention that patients received during their admission. Fifty-six per cent of these incidents were categorically classified as receiving only minimal medical intervention. On the other hand, 24/126 (19%) cases indicated that appropriate interventions were not done in a timely manner, leading to other infections and complications (further details are in Table 2).

**Table 2:** Reported quality of medical interventions

**Categorical Variable**	Example*	**Number of Incidents to offer this Experience**	**% Incidents to offer this Experience**
Minimal	Patient condition deteriorating. Doctors intervention minimal. Doctors not answering bleep.	71	56.35
Delayed	Admitted with acute septic arthritis of failed knee replacement and collapse at home while waiting revision. Subsequently died	24	19.05
Mistreated	Patient deteriorated over two days. Patient received possible sub-optimal care; observed not frequent enough, deterioration score not calculated correctly, lack of documentation and medical review, escalation not timely.	22	17.46
Inappropriate	A cardiac arrest call put out at 18.17, ALS procedure followed. Patient died. Examination of the medical notes and vital signs prior to this event reveal clear premonitory signs. Patient reviewed by SHO on 7/5/05 for hypotension. Gelofusin administered. It appears there is no further medical review until 9/5/05, time not stated. On the 8th at 22.00 the vital signs chart shows atrial fibrillation, rate 280. Vital signs show tachypnoea and persistent hypotension (Note seagull sign). Between 22.00 and 07.00 vital signs only done once, no time stated .No documented medical review.	9	7.14

*These reports are taken directly from the database and may be susceptible to grammatical errors.

### Skills of healthcare professionals

A failure of technical skills was identified in 32 cases. An example is given below:

"Patient became unresponsive with no pulse or respiratory effort. Arrest call put out. CPR [Cardiopulmonary resuscitation] commenced via [ambulatory] bag and cardiac massage. Response from arrest call was 2 staff nurses. The nurse carrying the arrest bleep informed them that she was ILS [immediate life support] trained but had not been updated for 5 years. The [doctor] appeared to be unaware of the function and working of the [defibrillator] machine and spent time trying to work out how to use it. It was suggested to him a number of times that he needed to secure an airway but he made no response to this. The machine then gave instructions to stand clear and press shock but no instructions were given by the [doctor] to move. 3 nurses had to ask the [doctor] to wait until everybody was clear. Switchboard [were] contacted to bleep the on call anaesthetist covering the ward. The anaesthetist who answered said that he did not cover [general wards] but he did speak to the SHO [senior house officer] [present]. CPR maintained during this. When the SHO returned he said that the anaesthetist would contact the Consultant anaesthetist. However no more contact was made. Further shock given by [doctor]. It was suggested to SHO that patient needed drugs. He stated he needed to wait as he had shocked the patient then he requested adrenaline and gave it. It was felt there was no support or anyone at the arrest with enough experience to co-ordinate the arrest. It was felt that someone who was competent in ALS [advanced life support] needed to ensure a co –ordinated event."

The second sub-category identified here was non-technical skills for surgeons (NOTSS). Among the four domains of NOTTS, as defined by Yule et al. as ‘*situational awareness, communication and teamwork, leadership and decision-making’*[18], the majority (51.7%) of the reported incidents can be classified under the domain of situational awareness. A further breakdown of these incidents is given in Table 3.

**Table 3:** NOTTS as a cause of death in surgical patients

**Domain**	**Frequency(n, %) N = 112**	**Example**
Situational awareness	58(51.8)	“Patient returned from theatre after NOF repair at 12.45. Vital signs at 14.15 show hypotension (seagull sign). No further observations recorded. At 14.30, nursing notes state the patient has not passed urine. Examination of the fluid balance chart suggests the patient has not passed urine at all that day. Did he in theatre? Not according to the anaesthetic chart. Fluid prescription chart shows 5 bags of fluid given not reflected on fluid balance chart[sic]”
Communication and teamwork	23(20.5)	“Patients conditions deteriorated at 1700 Dr P [staffname] informed. He attended to patient and tried to contact the orthopaedics team which he tried for four hours then to find he was not on call and was on holiday abroad. Patient’s condition deteriorated further. An anaesthetist was contact and saw patient.”
Leadership	18(16.1)	“Patient admitted with trauma to his right lower leg was administered anti-hypertensives and other medication prescribed for another patient. The patient’s condition deteriorated 6 hours later requiring transfer to critical care where he subsequently died approximately 38 hours following the medication error. No leadership on orthopaedics ward [sic]”
Decision-making	13(11.6)	“admitted for NOF repair, unwell from A& E, should have had 3 litres of fluid and 2 units of blood overnight with repeated ABGs at 4 pm. Overnight apparently unrecordable BP but no medical opinion was sought. 7 am ABGs done by HO. Condition worsening-attention then brought for low BP. SPR unsure [about] coming to ward…because of severity of illness and staffing levels. SPR called and central line inserted and IV fluids given. Patient died at 13.00 hrs RIP [sic]”

## Discussion

This is the first attempt to increase our knowledge and understanding of the burden of iatrogenic harm leading to mortality for the speciality of orthopaedics and trauma using a PSRS. However, this is only a start and much more needs to be done, given concerns about the utility of databases to promote safety. An increased rate of reporting, whereas in itself could imply that the culture of patient safety is improving, on its own is of limited value. The NPSA had received 158 incidents in 2003 and now has over five million incidents reported to it [19,20]. Paradoxically, despite the large number of incident reports received by the NPSA, reporting systems have been shown to detect only about 6% of adverse events found by systematic review of records [21]. Nevertheless, it is commendable that several solutions have been provided in the form of alerts and rapid responses [22].

We have shown that ethnographic techniques which are common place in qualitative research can be used to distil further learning from the database. Thematic analysis is one way to analyse qualitative information [23]. Our reviewers achieved substantial agreement (Kappa = 0.74) in the thematic analysis of the data [24]. This suggests that the corresponding framework created to better understand the typology and causality of the patient safety incidents was robust. Our approach has opted for themes not explored by other groups studying patient safety in orthopaedics: stages of the surgical journey, causality of iatrogenic harm leading to mortality, quality of medical interventions and skills of the healthcare professionals [25].

We have shown that almost three-quarters of the deaths in our study occurred outside the pre-operative phase. Similar findings were reported in a recent study by Cushner et al. that revealed that majority of the complications seen in patients undergoing arthroplasty of the hip or knee occur during the peri-operative (e.g. bleeding) and early post-operative period (e.g. deep vein thrombosis, wound infection, pneumonia) [17]. Several tools are now available to mitigate harm associated with poor care of orthopaedic patients, such as: pre- and post-operative adjuncts such as better use of orthogeriatric services [26]; early warning scores and trigger tools to prevent major catastrophes during pre-, intra- and post-operative phases of care [27]; enhanced recovery protocols [28] for the entire patient journey to ensure that best practice guidelines are adhered to; and intra-operative tools such as the WHO surgical checklist [29]. Yet we know that in some settings, like those found in England, uptake of these initiatives has been patchy [30]. A more concerted effort will have to be made by professional organisations to ensure that their members adhere to best practice guidelines to ensure safer care. The revalidation of healthcare professionals in the UK should also include domains that reflect the individual practitioner’s use of patient safety tools.

In our study, C. diff was frequently noted as a causative agent for mortality. This is unsurprising as the incidence and severity of C. difficile-associated diarrhoea has increased [31,32], Clostridium difficile in part due to antibiotic regimes that include cephalosporins, and also the demographics of the patients, who tend to be more elderly [33]. Greater collaboration between orthopaedic and microbiology departments should occur to ensure that local protocols are adhered to. Furthermore, healthcare-associated infections (HCAIs) are known to be the most frequent adverse event that threatens patient safety; as cited in the literature and within our study. The prevalence of these infections ranges from 5.7 and 19.1 per 100 inpatients. Furthermore, HCAIs can be broken down into surgical site infections (SSIs) (29%), urinary tract infections (24%), bloodstream infections (19%), healthcare-associated pneumonia (15%) and other infections (13%) [34]. The burden of these avoidable HCAIs is large; further steps are added to the patient’s journey that could include re-operation, extra nursing care and interventions, and further drugs. Fiscally, these factors have a significant bearing on any healthcare system [35]. Approaches to preventing SSIs come in three-phases: pre-, peri- and post-operative. Some pre-operative strategies include: patient showering and hair removal; patient and staff theatre wear; movement to and from theatre; nasal decontamination which does not involve routine use of mupirocin; mechanical bowel preparation; and antibiotic prophylaxis for specific groups of patients. Peri-operative measures include: hand decontamination; incise drapes; gowns and gloves; antiseptic skin preparation and diathermy; normal physiological parameters for patients (normal oxygenation, normoglycaemia, and normothermia); wound irrigation; and dressings and antiseptics before closure. Finally, in the post-operative phase, use should be made of: dressings; post-operative cleaning of surgical site; antibiotic treatment for SSI; and specialist wound care services [36].

In orthopaedic surgery, numerous attempts have been made to reduce SSIs in the operating theatre, including the use of peri-operative antibiotics, laminar flow operating rooms, body exhaust suits, multiple instrument trays and reduction of intra-operative operation room traffic [37-40]. Hand hygiene remains a key component in any infection prevention strategy. For many years, the traditional surgical scrub where the surgeon ensures that hands, nails and parts of the forearm are lathered and scrubbed has been standard practice. However, surgeons themselves accept that their practice, both in the operating theatre and outside, has often been suboptimal; 90% compliance is not good enough [41]. Some innovative solutions to the problem of SSIs include enhanced infection control initiatives [42] and multimodal quality improvement initiatives such as care bundles [43].

Our study highlights that almost half of all the deaths had elements of poor quality medical interventions. The highly-specialist nature of orthopaedic surgery means that surgeons are not always up-to-date or competent to deal with complex medical conditions which many patients, especially the elderly, present with. For example, it was suggested over 20 years ago that elderly patients undergoing orthopaedic surgery could benefit from input by geriatricians, owing to their comorbidities, frailty and reduction in independence [44-46]. It is only recently, however, that heightened political profiling through initiatives such as the new National Hip Fracture Database (NHFD), the Royal College of Physicians’ Audit of Falls and Bone Health, the Department of Health’s ‘Commissioning Toolkit’ and the National Institute for Health and Clinical Excellence’s (NICE) hip fracture guideline [47-50]. Furthermore, hip fracture is included in the ‘Best Practice Tariff’, which will financially reward units which include an orthogeriatrician in leading patient care [51]. There should be no excuse for unavoidable deaths due to poor medical management which falls outside the realm of the orthopaedic surgeon’s armamentarium.

One of the other key findings of our study was the large burden of a lack of non-technical skills which account for a significant proportion of iatrogenic harm. Almost 43% of all the deaths could be attributed to a lack of situational awareness, communication, teamwork and decision-making. It has been shown that most healthcare incidents can be attributed to failures in non-technical skills rather than technical ones [52]. Training in orthopaedic surgery has generally focused on clinical knowledge and expertise, including technical skills. There have been some attempts at introducing this type of training through various organisations such as the royal colleges [18]. However, greater effort is required to integrate non-technical skills into the educational activities of orthopaedic trainee doctors. Perhaps the momentum gained through the WHO surgical checklist, which aims to create well-functioning teams that improve the workings of the orthopaedic surgeon, will drive this agenda forward. Better teamwork and communication in operating theatres improves outcomes. Teamwork is definable and measurable, and can be improved through formal structured communication, such as checklists. Healthcare, and surgery in particular, is a team game, yet we have ignored the experiences of other high-risk industries to our patients’ cost. The WHO checklist and associated briefings and de-briefings are a major step forward in our approach to delivering the safe, reliable care we would want for our family, to all our patients [53].

### Limitations of the study and clinical relevance

One key limitation of this study is the inability to track anonymised incident reports back to their reporting hospitals so that further information can be obtained that would enable a deeper understanding of the error reports, which would have further enhanced our analysis. Other frequently cited biases include those related to reporting and hindsight [4]. Nevertheless, we feel that this paper should provide the impetus for greater clinical leadership in orthopaedic patient safety. Some advances have been made through use of checklists and integrated orthogeriatric services, for example, but an international focus is required to drive this agenda forward.

Another limitation relates to the NRLS database. The gross under-reporting to the database has been cited as its Achilles heel and, as such, its use is often limited to warning, communication and detection of rare patient safety incidents. Whilst this may be a valid criticism, it is clear that reporting is increasing as clinicians become more aware of its presence and, furthermore, develop confidence that there will not be any personal repercussions to making reports. Also, one might argue that a vast majority of incidents results in no harm whatsoever, which could create a false impression of over-reporting and the subsequent arguments of bureaucracy and misrepresentation of the situation. However, better and increased reporting of patient safety incidents will only further our quest for preventing all forms of avoidable harm in orthopaedic surgery.

## Conclusions

To our knowledge, this is the first attempt that has been made to make use of well-established qualitative methods to assess the burden of harm posed by orthopaedic patient safety incidents reported to a database of errors.

Iatrogenic harm in trauma and orthopaedic surgery is an important issue and we need a multi-pronged strategy to address it. In addition, to better study of the problem by building research capacity in the area, we need to act on known and proven interventions for delivering safer care; encourage better clinical leadership; promote the use of patient safety indicators as part of quality accounts for orthopaedic surgeons within hospitals; and showcase examples of best practice that use quality improvement and patient safety metrics.

## Abbreviations

ALS, Advanced life support; C. diff, Clostridium difficile; CPR, Cardiopulmonary resuscitation; HCAIs, Healthcare-associated infections; ILS, Immediate life support; IOM, Institute of Medicine; M&M, Morbidity and mortality; NHFD, National Hip Fracture Database; NHS, National Health Service; NICE, National Institute for Health and Clinical Excellence; NOTSS, Non-technical skills for surgeons; NPSA, National Patient Safety Agency; NRLS, National Reporting and Learning System; PSRS, Patient safety reporting systems; SHO, Senior house officer; SSI, Surgical site infection; WHO, World Health Organization.

## Competing interests

The authors declare that they have no competing interests.

## Authors’ contributions

All authors have made substantial contributions to the following: (1) the conception and design of the study, or acquisition of data, or analysis and interpretation of data; (2) drafting the article or revising it critically for important intellectual content; (3)final approval of the version to be submitted.

## Pre-publication history

The pre-publication history for this paper can be accessed here:

http://www.biomedcentral.com/1471-2474/13/93/prepub
